# A CRISPR Interference System for Efficient and Rapid Gene Knockdown in Caulobacter crescentus

**DOI:** 10.1128/mBio.02415-19

**Published:** 2020-01-14

**Authors:** Mathilde Guzzo, Lennice K. Castro, Christopher R. Reisch, Monica S. Guo, Michael T. Laub

**Affiliations:** a Department of Biology, Massachusetts Institute of Technology, Cambridge, Massachusetts, USA; b Howard Hughes Medical Institute, Massachusetts Institute of Technology, Cambridge, Massachusetts, USA; Johns Hopkins University School of Medicine; University of California, Berkeley

**Keywords:** CRISPR, Cas9, *Caulobacter crescentus*, gene knockdown

## Abstract

CRISPR interference (CRISPRi) is a powerful new tool used in different organisms that provides a fast, specific, and reliable way to knock down gene expression. Caulobacter crescentus is a well-studied model bacterium, and although a variety of genetic tools have been developed, it currently takes several weeks to delete or deplete individual genes, which significantly limits genetic studies. Here, we optimized a CRISPRi approach to specifically downregulate the expression of genes in C. crescentus. Although the Streptococcus pyogenes CRISPRi system commonly used in other organisms does not work efficiently in *Caulobacter*, we demonstrate that a catalytically dead version of Cas9 (dCas9) derived from the type II CRISPR3 module of Streptococcus thermophilus or from Streptococcus pasteurianus can each be effectively used in *Caulobacter*. We show that these CRISPRi systems can be used to rapidly and inducibly deplete *ctrA* or *gcrA*, two essential well-studied genes in *Caulobacter*, in either asynchronous or synchronized populations of cells. Additionally, we demonstrate the ability to multiplex CRISPRi-based gene knockdowns, opening new possibilities for systematic genetic interaction studies in *Caulobacter*.

## Abstract (precis)

Caulobacter crescentus is a major model organism for understanding cell cycle regulation and cellular asymmetry. The current genetic tools for deleting or silencing the expression of individual genes, particularly those essential for viability, are time-consuming and labor-intensive, which limits global genetic studies. Here, we optimized CRISPR interference (CRISPRi) for use in *Caulobacter*. Using Streptococcus thermophilus CRISPR3 or Streptococcus pasteurianus CRISPR systems, we show that the coexpression of a catalytically dead form of Cas9 (dCas9) with a single guide RNA (sgRNA) containing a seed region that targets the promoter region of a gene of interest efficiently downregulates the expression of the targeted gene. We also demonstrate that multiple sgRNAs can be produced in parallel to enable the facile silencing of multiple genes, opening the door to systematic genetic interaction studies. In sum, our work now provides a rapid, specific, and powerful new tool for silencing gene expression in C. crescentus and possibly other alphaproteobacteria.

## INTRODUCTION

Caulobacter crescentus is an oligotrophic alphaproteobacterium that serves as a major model organism for understanding the bacterial cell cycle and the origins of cellular asymmetry. Every cell division for C. crescentus produces two daughter cells with different cell fates (1, 2). One is a swarmer cell, which is motile, chemotactic, and unable to initiate DNA replication. The second is a stalked cell, which is sessile but competent for DNA replication. Swarmer cells can, given sufficient nutrients, differentiate into stalked cells, replacing their polar flagellum with a prosthetic stalk; coincident with this morphological transition, cells initiate DNA replication. Notably, C. crescentus cells will initiate replication once, and only once, per cell cycle under all known growth conditions leading to clearly defined G_1_, S, and G_2_ phases. This property has made C. crescentus an outstanding system for dissecting the molecular mechanisms that orchestrate cell cycle progression in bacteria. Additionally, the intrinsic polarity of cells and their obligate, asymmetric cell division have made C. crescentus a popular choice for investigating the origins and basis of cellular asymmetry, a common feature in the life cycles of many bacteria and virtually all eukaryotes.

Although C. crescentus is genetically tractable and a large arsenal of genetic tools has been developed (3, 4), it remains rather laborious and time-consuming to generate a deletion strain or, for genes essential for viability, a strain in which the gene of interest is placed under the control of a regulated promoter to enable transcription-based depletion. Current approaches that rely on recombination with long regions of homology take ∼2 to 3 weeks.

New tools for knocking down the expression of individual genes in a variety of organisms have been developed in recent years using CRISPR (clustered regularly interspaced short palindromic repeats) and the associated protein Cas9 (5). CRISPR systems are naturally found in ∼50% of all bacteria (6), where they help cells prevent infection by some bacteriophage (7, 8). CRISPR loci contain a series of repeat sequences with intervening protospacers derived from phage. For type II CRISPR systems, the protospacers and repeats are expressed as a single RNA, with individual spacers then cut out and loaded into a Cas9 protein along with a tracer RNA (9). This loaded Cas9 can then recognize incoming phage DNA that harbors a perfect match to the spacer RNA and that contains a protospacer adjacent motif (PAM) just downstream of the targeted region. Cas9 then cleaves incoming phage DNA, thereby thwarting an infection (7, 10). The requirement for a PAM, which is not present in the CRISPR locus itself, prevents chromosome cleavage and self-intoxication by Cas9.

The CRISPR-Cas9 system has been repurposed to enable facile site-specific genome engineering. Expression of Cas9 and a single guide RNA (sgRNA), which combines the CRISPR RNA (crRNA) and the transactivating crRNA (tracrRNA) into a single RNA, is sufficient in most organisms to drive DNA cleavage at a defined locus (11, 12). This site-specific cleavage can be coupled with homologous recombination-based repair of the cleaved site to enable the introduction of a desired DNA construct. Alternatively, an enzymatically dead variant of Cas9, referred to as dCas9, can be used to target Cas9 to specific genomic loci to block transcription, but without DNA cleavage (5, 13). Such dCas9-driven transcriptional knockdowns, or CRISPR interference (CRISPRi) systems, are generally more efficient with guide RNAs complementary to the nontemplate strand and positioned toward the 5′ end of a transcribed region (5, 14).

CRISPRi systems have been developed and applied for several bacteria, including Bacillus subtilis, where CRISPRi has enabled powerful, genome-wide knockdown studies (15). For B. subtilis, and for many other organisms, the dCas9 enzyme used derives from Streptococcus pyogenes. However, for some organisms, such as Mycobacterium smegmatis, high expression of *dcas9* from S. pyogenes is lethal and lower expression levels lead to poor CRISPRi knockdown efficiency (16). Screening of Cas9 orthologs from other *Streptococcus* species led to the identification of five additional type IIA Cas9 enzymes that were efficient for CRISPR-based DNA cleavage and CRISPRi gene knockdown studies in M. smegmatis and Mycobacterium tuberculosis (16).

**FIG 1:**
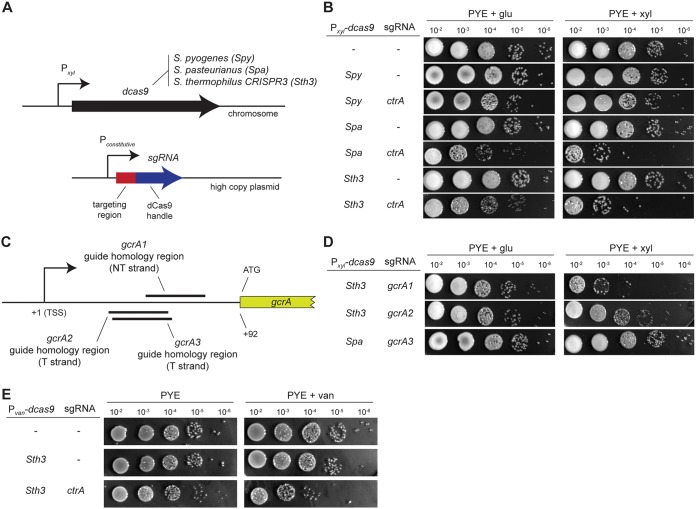
CRISPRi systems to downregulate gene expression in Caulobacter crescentus. (A) Design of the different constructs used in this study. Each system consists of a nuclease-dead variant of the *cas9* gene (*dcas9*) expressed under the control of a xylose (or vanillate) promoter and the corresponding specific single guide RNA (sgRNA) constitutively expressed (see Materials and Methods). (B) Efficiency of downregulating *ctrA* expression using the different CRISPRi systems from S. pyogenes, S. thermophilus CRISPR3, and *S. pasteurianus*. Shown are serial dilutions of each strain on PYE supplemented with 0.2% glucose or 0.3% xylose after 48 h at 30°C. Note that the commonly used S. pyogenes system is not functional in C. crescentus, although it targets the same sequence as the *S. pasteurianus* system here. Strand and sequence targeting of the different sgRNAs is detailed in Fig. S1A. (C) Strand and sequence targeting of the different sgRNAs for *gcrA* used in panel D. TSS, transcription start site; T, template; NT, nontemplate. (D) Efficiency of downregulating *gcrA* expression when targeting the nontemplate strand using dCas9 derived from S. thermophilus CRISPR3 and *S. pasteurianus*. Shown are serial dilutions of each strain on PYE supplemented with 0.2% glucose or 0.3% xylose after 48 h at 30°C. (E) Efficiency of downregulating *ctrA* expression with the dCas9 of S. thermophilus CRISPR3 under a vanillate-inducible promoter. Shown are serial dilutions of each strain on PYE or PYE supplemented with 500 μM vanillate after 48 h at 30°C.

For Caulobacter crescentus, which does not encode a native CRISPR/Cas system (17), the conventional S. pyogenes dCas9 was reported to work for CRISPRi gene knockdown (18). However, we were unable to achieve detectable silencing of target genes with this enzyme in C. crescentus (see below, Fig. 1) but found that two Cas9 orthologs—from Streptococcus thermophilus and Streptococcus pasteurianus—enabled highly efficient gene knockdown. We report here the development of these CRISPRi systems and demonstrate their efficacy in depleting two well-characterized, essential cell cycle regulators, CtrA and GcrA. The depletion of these factors by CRISPRi led to phenotypes comparable to those seen with temperature-sensitive and transcriptional depletion strains, with similar kinetics. We also demonstrate the use of the S. thermophilus CRISPRi system for the simultaneous knockdown of two genes, opening the door to systematic genetic interaction studies. In sum, the CRISPRi systems developed here should see wide applicability in studies of C. crescentus and possibly other, related alphaproteobacteria.

## RESULTS

### Design and development of a CRISPRi system for C. crescentus.

To develop a CRISPRi system for Caulobacter crescentus, we tested dCas9 proteins derived from three different species, which have all been used previously for CRISPRi knockdowns: Streptococcus pyogenes, Streptococcus thermophilus CRISPR3, and Streptococcus pasteurianus. We cloned *dcas9* from each organism (16) downstream of a xylose-inducible, glucose-repressible promoter in the pXGFPC-5 nonreplicative plasmid (3) (Fig. 1A). This plasmid contains a region of homology to the endogenous *xylX* locus found in the *Caulobacter* genome, enabling stable, chromosomal integration at the *xylX* locus, which we verified by PCR. To test if induction of each dCas9 variant alone affects the growth of C. crescentus, we performed serial dilutions on peptone-yeast extract (PYE) plates supplemented with glucose or xylose to repress or induce, respectively, *dcas9* expression. For each strain carrying *dcas9*, growth on 0.2% glucose or induction with 0.3% xylose did not lead to any detectable reduction in viability compared to a wild-type control strain (Fig. 1B), indicating that dCas9 alone does not significantly affect the growth or fitness of C. crescentus.

10.1128/mBio.02415-19.1 FIG S1 Controls for assessing CRISPRi systems to downregulate gene expression in Caulobacter crescentus. (A) Strand and sequence targeting of the different *ctrA*-sgRNAs used in Fig. 1B. TSS, transcription start site; T, template; NT, nontemplate. (B) Serial dilutions of the *ctrA401^ts^* control spotted on PYE plates incubated for 48 hours at 30°C or 37°C where indicated. (C) Expression of the sgRNAs targeting *ctrA* or *gcrA* do not affect cell viability in cells lacking the dCas9 protein. Shown are spotting of serial dilutions of each strain on PYE supplemented with 0.2% glucose or 0.3% xylose after 48 hours at 30°C. (D) Serial dilutions of the Δ*gcrA* P*_van_-gcrA* strain spotted on PYE plates supplemented with 500 μM vanillate where indicated and incubated for 48 hours at 30°C. Download FIG S1, TIF file, 2.6 MB. Copyright © 2020 Guzzo et al. 2020 Guzzo et al. This content is distributed under the terms of the Creative Commons Attribution 4.0 International license.

To test the ability of each dCas9 to downregulate gene expression, we generated single guide RNAs (sgRNAs) composed of a 20-nucleotide (nt) targeting region followed by the species-specific crRNA direct repeat and tracrRNA (11, 16), which includes the dCas9 handle and a transcriptional terminator. These sgRNAs were placed on a replicative high-copy-number plasmid, pBXMCS-2 (3), under the control of a constitutive promoter (Fig. 1A; see Materials and Methods). To test the efficiency of each CRISPRi system, we targeted an essential and well-studied gene, *ctrA*, which encodes a cell cycle-regulated master transcription factor in *Caulobacter.* Given previous studies showing that targeting of the nontemplate strand of a gene’s promoter or coding region is most efficient for CRISPRi-mediated gene silencing (5, 14), we generated sgRNAs that target the nontemplate strand of *ctrA* between the transcription start site and the coding region, with the exact location dependent on the location of species-specific PAMs for each dCas9 (see Fig. S1A in the supplemental material).

Because *ctrA* is essential for viability (Fig. S1B), an effective CRISPRi system targeting *ctrA* should prevent, or significantly diminish, growth on plates following *dcas9* expression. As a control, we used a *ctrA* temperature-sensitive mutant (*ctrA401^ts^* [19, 20]), in which the CtrA protein gets inactivated at high temperatures, leading to a strong growth defect when cells are grown at 37°C instead of the usual 30°C (Fig. S1B). To test if CtrA was depleted after expressing each *dcas9* and the corresponding sgRNA, we first performed serial dilutions on PYE plates supplemented with glucose or xylose to repress or induce, respectively, dCas9. For the CRISPRi systems from S. thermophilus CRISPR3 and *S. pasteurianus*, we observed an ∼1,000-fold reduction in growth when dCas9 was induced. The downregulation of *ctrA* required dCas9; inducing only the *ctrA*-sgRNA did not affect growth (Fig. S1C). Notably, the CRISPRi system derived from S. pyogenes did not affect growth (Fig. 1B), even when the targeted sequence was identical to that used for *S. pasteurianus* (Fig. S1A), indicating that S. pyogenes dCas9 in our hands does not effectively silence genes in C. crescentus. We conclude that the dCas9 enzymes from S. thermophilus CRISPR3 and *S. pasteurianus* are both effective for CRISPRi in C. crescentus.

To test a second gene and to determine the ideal region of a transcript to target for downregulating expression, we designed sgRNAs targeting different positions on the template and the nontemplate strands of the *gcrA* transcript adjacent to PAM sites. Like CtrA, GcrA is an essential transcription factor in *Caulobacter*; a depletion strain in which *gcrA* is expressed from a vanillate-inducible promoter shows severely diminished growth in the absence of vanillate (21) (Fig. S1D). As with our experiments targeting *ctrA*, we measured the efficiency of different sgRNAs in silencing *gcrA* by serial dilutions of cultures on PYE plates supplemented with glucose or xylose to repress or induce, respectively, the S. thermophilus dCas9 (Fig. 1C and D). We observed an ∼1,000-fold reduction of growth only when the guide sequence was complementary to the nontemplate strand of the leader region of the *gcrA* transcript (Fig. 1C and D) between the transcription start site and the coding region. Similarly, we found that the *S. pasteurianus* CRISPRi system inhibited growth when targeting *gcrA* or *ctrA* only when the sgRNA was complementary to the nontemplate strand between the transcriptional start site and the start codon (Fig. 1B to D; Fig. S1A).

For both the S. thermophilus CRISPR3i and *S. pasteurianus* CRISPRi systems, we observed an ∼10-fold decrease in plating efficiency relative to the wild type under noninducing conditions, likely reflecting leaky expression of *dcas9* (Fig. 1B). To tighten the regulation of *dcas9* expression, we tested a vanillate-inducible promoter commonly used in studies of C. crescentus (3) to regulate *dcas9* expression. To do so, we cloned the *dcas9* gene from S. thermophilus CRISPR3i into the pVCERC-5 plasmid, transformed it into C. crescentus, and selected for integration at the vanillate locus. In the absence of a guide RNA, induction of *dcas9* with 500 μM vanillate did not affect growth as tested by serial dilutions (Fig. 1E). We then combined the vanillate-inducible *dcas9* with the *ctrA*-sgRNA and observed a very small effect on growth under noninducing conditions, suggesting that the vanillate-inducible system is less leaky than the xylose one. However, under inducing conditions, cells harboring *dcas9* and the *ctrA-sgRNA* exhibited an ∼100-fold reduction of growth (Fig. 1E). We conclude that the *van* promoter provides tighter regulation of *dcas9* expression than the *xyl* promoter but somewhat weaker expression upon maximum induction.

Because the PAM sequence of S. thermophilus CRISPR3, which is NGGNG, is more commonly found in the GC-rich C. crescentus genome (264,782 NGGNG PAM sequences) than the PAM sequence from *S. pasteurianus*, which is NNGTGA (25,334 NNGTGA PAM sequences), we focused primarily on the S. thermophilus CRISPR3 (*Sth3*) system for the rest of this study. Additionally, we chose to use the xylose-inducible CRISPRi system which is leaky but enables stronger knockdown than the vanillate-inducible system.

### CRISPRi rapidly shuts off expression of target genes.

To further characterize the efficiency of the *Sth3* CRISPRi system, we measured the timing of CtrA and GcrA depletion in a mixed, asynchronous population of cells using sgRNAs specific to each transcript targeting the nontemplate strand (Fig. 1C; Fig. S1A). In each case, we grew cells to exponential phase in a rich medium supplemented with glucose and then split the culture, without washing out the glucose, adding 0.3% xylose to one culture to induce the CRISPRi system while keeping the other in glucose to continue repression of *dcas9*. When targeting *ctrA*, we harvested samples every 20 min from the noninduced (glucose) and induced (glucose plus xylose) cultures for 120 min. Because the GcrA protein is stable in stalked cells, when targeting *gcrA* we extended the time course to 240 min, with sampling every 40 min, to achieve full depletion of the protein. The CRISPRi strains targeting *ctrA* or *gcrA* were each compared to a control strain that lacks an sgRNA but still contains P*_xyl_*-*dcas9* on the chromosome.

**FIG 2:**
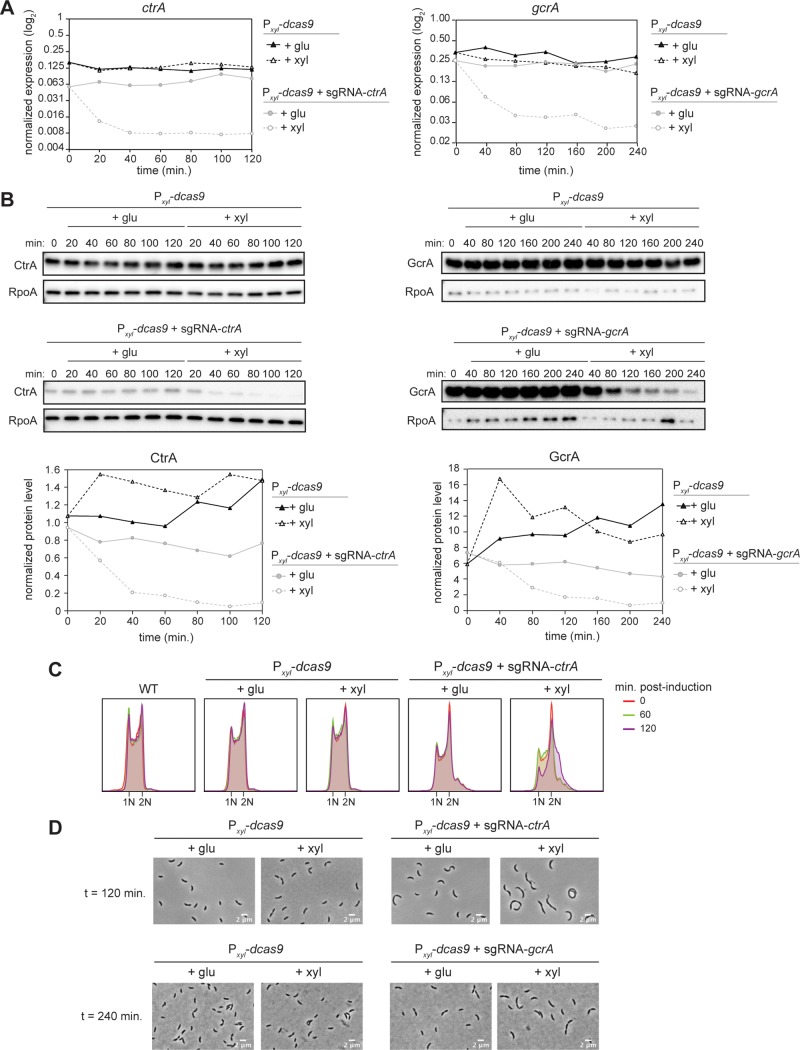
Downregulation of *ctrA* and *gcrA* expression in asynchronous populations of cells using the *Sth3* CRISPRi system. (A) *ctrA* (left) and *gcrA* (right) mRNA levels measured by qRT-PCR and normalized to *rpoA* levels. (B) CtrA (left) and GcrA (right) protein levels with and without induction of the CRISPRi system. Graphs show quantifications of CtrA or GcrA protein band intensity normalized to RpoA protein band intensity. (C) Flow cytometry profiles showing the DNA content of a mixed population of cells at different time points after induction or not of the *ctrA*-targeting CRISPRi system. (D) Phase-contrast images of cells 120 (top) or 240 (bottom) min after induction or not of *dcas9* alone or in combination with the *ctrA*- or *gcrA*-sgRNA, as indicated. Bar, 2 μm.

To measure changes in transcript levels, we used qRT-PCR, comparing *ctrA* and *gcrA* to a control locus, *rpoA*. For both *ctrA* and *gcrA*, expression under inducing and noninducing conditions was already slightly reduced in the strain containing the sgRNA relative to the control strain at the start of the experiment (Fig. 2A) due to leaky expression of the CRISPRi system, consistent with our plating viability results (Fig. 1B). In the strain expressing both *dcas9* and the *ctrA*-sgRNA, the levels of *ctrA* mRNA decreased drastically after just 20 min in xylose and then remained low throughout the rest of the time course, demonstrating efficient and rapid downregulation of *ctrA* expression (Fig. 2A). When this strain was maintained in glucose, the expression of *ctrA* remained relatively constant. For the strain expressing *dcas9* and the *gcrA-*sgRNA, *gcrA* expression decreased significantly after 40 min and reached a low plateau within 80 min (Fig. 2A). Again, maintaining this strain in glucose led to relatively constant levels of *gcrA*. In contrast to the strains targeting *ctrA* and *gcrA*, the strain harboring only dCas9 exhibited relatively constant expression of *ctrA* and *gcrA* throughout the time courses, with or without induction (Fig. 2A).

We also observed lower CtrA and GcrA protein levels in the strains that contain the respective sgRNA even without induction of dCas9 (Fig. 2B). Following dCas9 induction, CtrA protein levels were severely reduced after 40 min and almost undetectable after 60 min (Fig. 2B), reflecting efficient inhibition of *ctrA* transcription and the relatively short half-life of CtrA protein (22). GcrA protein also decreased rapidly following dCas9 induction, though not as fast as CtrA, likely because GcrA is a more stable protein (Fig. 2B). In the strain containing only dCas9, there was no significant change in CtrA or GcrA levels throughout the time courses, with or without induction (Fig. 2B). In sum, our results indicate that the *Sth3* CRISPRi system leads to a rapid inhibition of target gene expression, with maximal transcriptional inhibition occurring within a single generation.

### CRISPRi knockdown of *ctrA* and *gcrA* phenocopies known, loss-of-function mutants.

To test whether our CRISPRi system produces the known, loss-of-function phenotypes for cells lacking *ctrA*, we first used flow cytometry to compare cells harboring the *ctrA*-sgRNA to a well-characterized *ctrA401^ts^* allele (19, 20). Because CtrA is required for cell division and inhibits new rounds of DNA replication, cells lacking CtrA accumulate more than two chromosomes (19). Even when grown at the permissive temperature of 30°C, some *ctrA401^ts^* cells had more than 2 chromosomes (Fig. S2A). Similarly, in the strain containing dCas9 and the *ctrA-*sgRNA, a small proportion of cells had more than 2 chromosomes in the absence of induction (Fig. 2C). Hence, both depletion systems are leaky in the absence of induction. When shifted to the restrictive temperature of 37°C, the flow cytometry profile for *ctrA401^ts^* cells rapidly shifted toward higher chromosome content with a significant decrease in 1N cells, as expected (Fig. S2A). The flow cytometry profile for the strain harboring *dcas9* and the *ctrA-*sgRNA also showed a decrease in 1N cells and a shift toward increased chromosome content under the induced condition (Fig. 2C). The changes were less pronounced than with the *ctrA401^ts^* strain (Fig. S2A), likely because the shift to a restrictive temperature for a *ctrA401^ts^* strain leads to a rapid loss in protein function whereas the CRISPRi strain blocks transcription and therefore relies on proteolysis and dilution to eliminate CtrA. As CtrA is unstable during only a short window of time during the cell cycle (22), a loss of CtrA and consequent change in DNA content take longer to manifest with CRISPRi.

10.1128/mBio.02415-19.2 FIG S2 Loss-of-function phenotypes for cells lacking CtrA or GcrA. (A) Flow cytometry profiles showing the DNA content of a mixed population of *ctrA401^ts^* cells at different time points. *t* = 0 corresponds to when cells were split for growth at 30 and 37°C. (B) Phase-contrast images of cells 120 min after shifting to 37°C for the *ctrA401^ts^* strain (top) or after washing away the vanillate for the Δ*gcrA P_van_-gcrA* strain (bottom), as indicated, leading to cell elongation in both cases. Bar, 2 μm. Download FIG S2, TIF file, 2.3 MB. Copyright © 2020 Guzzo et al. 2020 Guzzo et al. This content is distributed under the terms of the Creative Commons Attribution 4.0 International license.

Because a loss of CtrA or GcrA leads to cell elongation (Fig. S2B), we also looked at cell morphology using phase-contrast microscopy after 120 min (for *ctrA*) or 240 min (for *gcrA*) of dCas9 induction. For CtrA, we observed that cells expressing the CRISPRi system became elongated, relative to a strain expressing only dCas9 (Fig. 2D), as also seen for the *ctrA401^ts^* mutant following a shift to the restrictive temperature (Fig. S2B). Similarly, when targeting *gcrA* with the CRISPRi system, cells were clearly elongated after 240 min of CRISPRi induction (Fig. 2D) with cell morphology comparable to that seen after 2 h with a GcrA-depletion strain (Δ*gcrA P_van_-gcrA*) (21) (Fig. S2B).

**FIG 3:**
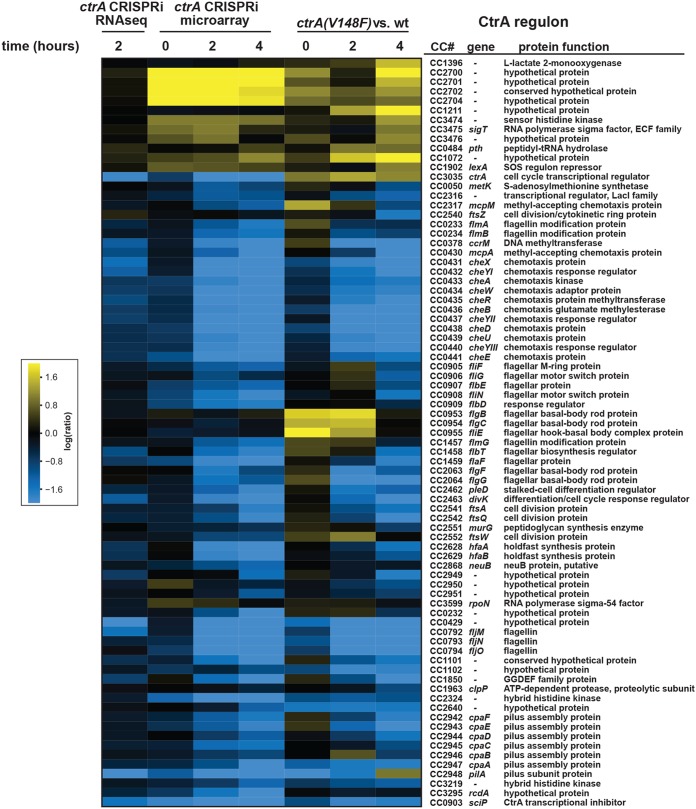
Repression of *ctrA* expression by CRISPRi leads to misregulation of CtrA-regulated genes. Expression changes of CtrA-regulated genes in cells expressing *dcas9* and the *ctrA*-sgRNA (+ xyl) compared to the noninducing condition (+ glu). Column 1, after 2 h by RNA-seq; columns 2 to 4, at *t* = 0, after 2 h, or after 4 h by microarray. As a control, expression changes of CtrA-regulated genes in the *ctrA*(*V148F*)*^ts^* strain compared to wild type (wt) are shown in columns 5 to 7 (23, 24).

To determine if the CRISPRi system targeting *ctrA* leads to changes in the entire CtrA regulon, and to determine whether CRISPRi has any potential off-target effects, we used RNA sequencing (RNA-seq) and DNA microarrays to examine global patterns of gene expression. We first used DNA microarrays to examine the strain containing dCas9 and the *ctrA-*sgRNA at 0, 2, and 4 h after induction of the sgRNA, with the fold change in gene expression at each time point reporting the ratio in induced (+ xylose [xyl]) to noninduced (+ glucose [glu]) cells. We then compared the expression changes in known CtrA-regulated genes in the dCas9 plus *ctrA-*sgRNA strain and a *ctrA*(*V148F*)*^ts^* strain following a shift to a restrictive temperature (23, 24). The genes in the CtrA regulon showed similar changes in the two strains, in terms of the magnitude and timing of the changes (Fig. 3). We also performed RNA-seq on the strain containing dCas9 and the *ctrA*-sgRNA, comparing gene expression changes 2 h after induction of *dcas9*. Again, genes in the CtrA regulon, particularly those that are activated by CtrA, showed similar changes in the CRISPRi strain as seen previously in the *ctrA*(*V148F*)*^ts^* strain (Fig. 3).

To assess potential off-target effects of the *Sth3* CRISPRi system, we considered the 40 genes showing the largest change in expression following induction of the *ctrA*-sgRNA (Table S1 and Fig. S3). These 40 genes represent the major gene expression changes observed by RNA-seq in the strain containing the *ctrA*-sgRNA compared to the *dcas9*-only strain when comparing the induced and noninduced conditions (Fig. S3). Of these 40 genes, only 20 also had data in our microarray experiment and all 20 were similarly downregulated. We also found that 32 of these 40 most highly affected genes were similarly affected in previous microarray data generated for the *ctrA*(*V148F*)*^ts^* strain (23, 24), and 38 were identified by ChIP-seq studies as likely direct targets of CtrA (25, 26) (Table S1). The absence of other major changes in gene expression that are not attributed to a decrease in CtrA indicates that the CRISPRi system does not lead to significant or obvious off-target effects on transcription. Taken all together, our gene expression studies indicated that the CRISPRi system developed here is efficient and specific in knocking down target gene function.

10.1128/mBio.02415-19.3 FIG S3 Off-target effects of the *Sth3* CRISPRi system. Log_2_ fold changes in gene expression for the *ctrA*-sgRNA strain in xylose versus glucose compared to the *dcas9*-only strain in xylose versus glucose. The effects on *ctrA* (pink) and the 40 genes that exhibit the largest gene expression changes following induction of *ctrA*-sgRNA (orange) are shown (Table S1). The dashed line demarcates where gene expression changes are equal between the *ctrA*-sgRNA and the *dcas9*-only strain. Genes that are induced or repressed in the *ctrA*-sgRNA strain but lie along the dashed line are genes whose expression changes are dependent on the shift from glucose to xylose. Download FIG S3, TIF file, 2.7 MB. Copyright © 2020 Guzzo et al. 2020 Guzzo et al. This content is distributed under the terms of the Creative Commons Attribution 4.0 International license.

10.1128/mBio.02415-19.4 TABLE S1 Top 40 most affected genes following CRISPRi downregulation of *ctrA*. Download Table S1, XLSX file, 0.02 MB. Copyright © 2020 Guzzo et al. 2020 Guzzo et al. This content is distributed under the terms of the Creative Commons Attribution 4.0 International license.

### CRISPRi knockdown of *ctrA* and *gcrA* in synchronized cells.

**FIG 4:**
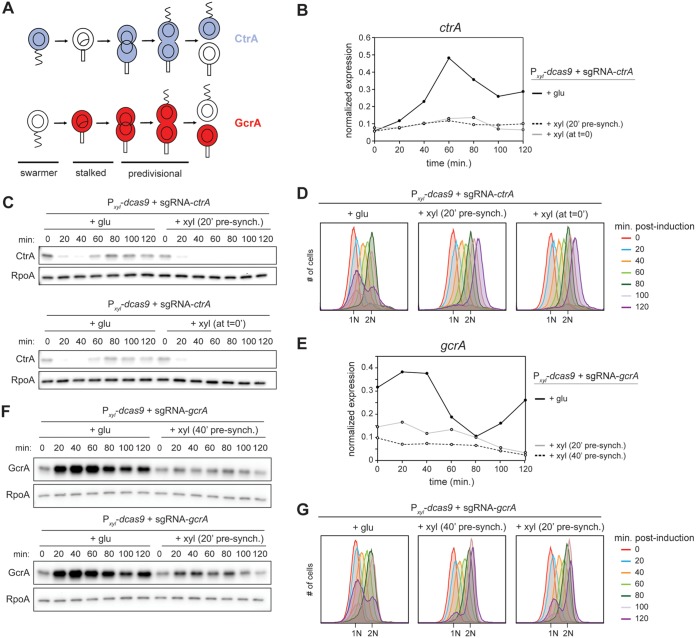
Downregulation of *ctrA* and *gcrA* expression in synchronized cells. (A) Schematic of CtrA and GcrA abundance during the *Caulobacter* cell cycle. (B) Normalized *ctrA* expression in synchronized cells after induction of the CRISPRi system at *t* = 0 min after synchronization or *t* = 20 min before synchronization. (C) CtrA protein levels in synchronized cells after induction of CRISPRi system at the same times as indicated for panel B. (D) Flow cytometry profiles after SYTOX staining showing DNA content of synchronized cells when targeting *ctrA*. (E) Normalized *gcrA* expression in synchronized cells after induction of the CRISPRi system at *t* = 20 or 40 min before synchronization. (F) GcrA protein levels in synchronized cells after induction of CRISPRi system at the same times as indicated for panel E. (G) Flow cytometry profiles after SYTOX staining showing DNA content of synchronized cells when targeting *gcrA*.

*Caulobacter* is a major model organism for studying the bacterial cell cycle in large part because a population of cells can be easily synchronized by density centrifugation. Once isolated, the G_1_ swarmer cells can be released into fresh medium to follow cell cycle progression. We tested the efficiency of the CRISPRi system for depleting CtrA and GcrA in synchronized populations of cells. Notably, CtrA and GcrA have opposite patterns of abundance during the cell cycle (Fig. 4A), with GcrA maximally abundant in stalked cells and early predivisional cells, while CtrA levels peak in late predivisional and swarmer cells. For *ctrA*, after washing out the glucose, we induced the CRISPRi system either 20 min before synchronizing cells or when releasing the cells into fresh medium after synchronization. Using qRT-PCR, we detected a small, residual increase in *ctrA* expression at 60 to 80 min postsynchronization, when inducing the CRISPRi system at 0 min (Fig. 4B). However, induction 20 min before synchronization completely abolished the expression of *ctrA* throughout the cell cycle (Fig. 4B). Under both conditions, CtrA protein levels were very low 60 min postsynchronization compared to the dCas9-only control strain (Fig. 4C). Flow cytometry analysis of chromosome content showed that, when inducing dCas9 at *t* = 0, or 20 min before synchronization, we observed virtually no new 1N cells at *t* = 120 min (Fig. 4D), indicating a nearly complete loss of CtrA activity in both conditions.

We also tested GcrA depletion in synchronized cells. *gcrA* expression normally increases rapidly after synchronization, reaching a maximum after ∼20 min (Fig. 4E). To inhibit the expression of *gcrA* in a synchronized population of cells, we washed the cells to remove glucose and tested induction of the CRISPRi system 20 and 40 min before synchronization. Induction 40 min before synchronization was most efficient, with very little *gcrA* transcription (Fig. 4E) and almost no GcrA protein present at the end of the first cell cycle (Fig. 4F). Consistent with a substantial reduction in GcrA, the proportion of cells that divided to yield new, 1N cells, as detected by flow cytometry, was strongly reduced relative to the no-induction condition (Fig. 4G).

Taken all together, our results indicate that the *Sth3* CRISPRi system developed here is efficient at downregulating genes in synchronized populations of cells. The precise timing of protein loss depends on the stability and cell type distribution of a given protein during the cell cycle, but this CRISPRi system should offer wide utility.

### Dual targeting with CRISPRi.

Finally, we tested whether two genes could be simultaneously downregulated using the xylose-inducible *Sth3* CRISPRi system. We chose to combine downregulation of the *cpaA* and *blaA* genes, which encode a prepilin peptidase, part of the pilus apparatus, and a β-lactamase, respectively. The null phenotypes of cells lacking these two genes are easily scored. In *Caulobacter*, the phage ΦCbK uses pili as receptors to infect the cells (27). Hence, pilus mutants, including a *cpaA* mutant, are resistant to ΦCbK. The β-lactamase encoded by *blaA* has been shown to confer *Caulobacter*’s natural resistance to carbenicillin (28).

**FIG 5:**
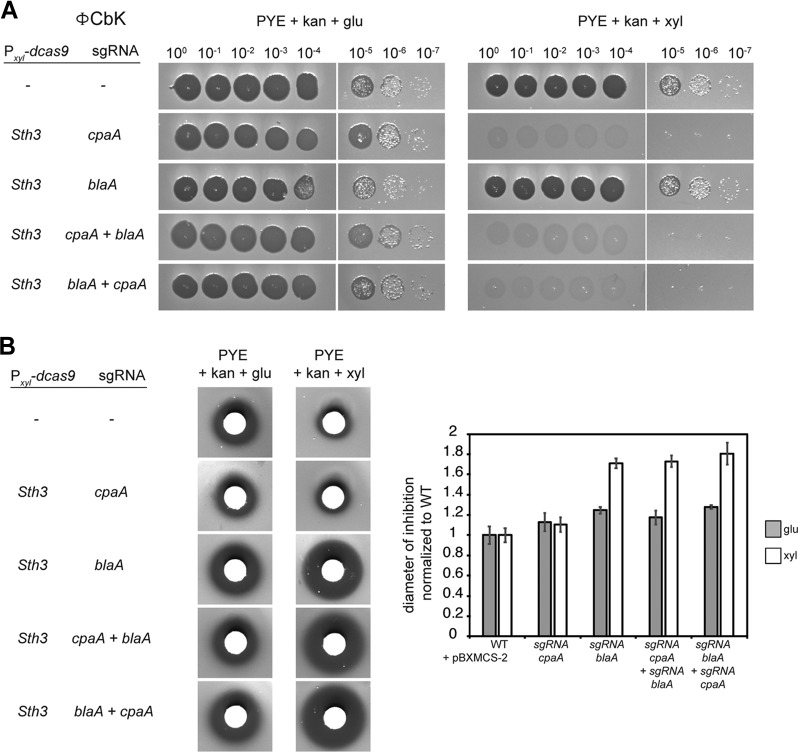
Dual targeting of *cpaA* and *blaA* with CRISPRi. (A) Assay to assess sensitivity to phage ΦCbK. Downregulation of the prepilin peptidase gene *cpaA* confers resistance to the ΦCbK. Phage dilutions were spotted on a lawn of C. crescentus on PYE supplemented with kanamycin and 0.2% glucose or 0.3% xylose. (B) Growth inhibition assay to assess sensitivity to carbenicillin. Downregulation of the β-lactamase gene *blaA* enhances sensitivity to carbenicillin. Pictures on the left show the zone of growth inhibition (dark zone) surrounding carbenicillin-soaked disks for each indicated strain grown on PYE supplemented with kanamycin and 0.2% glucose or 0.3% xylose. The graph on the right represents the average diameter of inhibition normalized to wild type (WT), measured on *n* = 6 replicates from *n* = 2 biological replicates for each strain.

When expressing *dcas9* with a *cpaA*-sgRNA, we observed strong resistance to the ΦCbK phage, as expected, only when inducing the CRISPRi system (Fig. 5A). The *cpaA* CRISPRi strain retained resistance to carbenicillin, comparable to the wild type (Fig. 5B). Thus, the *cpaA-*sgRNA CRISPRi system is efficient at specifically reducing *cpaA* expression. In contrast to *cpaA*, the CRISPRi system targeting *blaA* had no effect on ΦCbK phage sensitivity relative to the wild type (Fig. 5A) but significantly increased the diameter of growth inhibition around the carbenicillin-soaked disks (Fig. 5B), indicating a reduction in carbenicillin resistance.

As *cpaA* and *blaA* CRISPRi knockdowns have clearly distinguishable phenotypes, we combined the two sgRNAs in the same plasmid to simultaneously downregulate the expression of both genes. We cloned the entire locus containing *blaA-*sgRNA and its constitutive promoter downstream (*cpaA* + *blaA*) or upstream (*blaA* + *cpaA*) from the *cpaA-*sgRNA, so that each guide had its own promoter. When inducing *dcas9_Sth3_* in the presence of either dual-targeting plasmid, we observed (i) an increase in carbenicillin sensitivity comparable to that observed when targeting *blaA* alone (Fig. 5B) and (ii) resistance to ΦCbK comparable to cells targeting *cpaA* alone (Fig. 5A). Similar results were obtained regardless of the order in which the sgRNAs were cloned. We conclude that the CRISPRi system can be used to downregulate the expression of multiple genes in the same strain in *Caulobacte*r, which is currently a laborious and time-consuming process. Thus, the CRISPRi system designed here is a powerful new tool for studying genetic interactions in C. crescentus.

## DISCUSSION

We developed a CRISPRi system for the specific and efficient inhibition of transcription in Caulobacter crescentus. Although CRISPR-based knockdown and genome editing systems have become relatively common in studies of eukaryotes, they remain relatively underused in studies of bacteria. In particular, to the best of our knowledge, a CRISPRi system has not been reported for an alphaproteobacterium. One study reported use of an S. pyogenes-derived dCas9 in Caulobacter crescentus (18), but at least in our hands, dCas9 from S. pyogenes does not work efficiently in *Caulobacter*. Similarly, the S. pyogenes dCas9 is not very efficient in Mycobacterium smegmatis (16). However, several Cas9 orthologs from other streptococci were effective in M. tuberculosis (16). Two of these, one from S. thermophilus and one from *S. pasteurianus*, also worked effectively in *Caulobacter*.

Our CRISPRi system involves the constitutive expression of a single guide RNA targeting a transcript of interest with the dCas9 driven by an inducible promoter. The kinetics of gene knockdown are thus driven primarily by the speed at which dCas9 accumulates. The onset of a consequent phenotype also depends on how quickly the protein produced by a given transcript decays, as with any transcription-based depletion system. For proteins like CtrA, which is completely degraded during the G_1_-S transition, the phenotype emerges within a single generation. A comparison of our CRISPRi-based knockdown of *ctrA* and a well-studied temperature-sensitive strain revealed similar changes in gene expression after 2 and 4 h of dCas9 induction and a shift to the restrictive temperature, respectively (23, 24) (Fig. 3). For other proteins like GcrA, which remains relatively stable once it accumulates in stalked cells, the onset of a phenotype following dCas9 induction is slower because it depends more on dilution occurring over multiple generations. In this regard, all CRISPRi systems are inferior to fast-acting, temperature-sensitive alleles, but CRISPRi has the advantage, of course, of being rapidly implemented for any gene of interest and of avoiding the pleiotropic effects of a temperature shift.

To knock down the expression of genes using CRISPRi in *Caulobacter*, we conditionally expressed *dcas9* from either a vanillate- or a xylose-inducible promoter. The xylose-inducible expression system yields stronger repression in the presence of a specific sgRNA but is leakier in the absence of induction. Despite the leakiness, we were able to efficiently use and characterize the downregulation of essential genes like *ctrA* and *gcrA*. However, if the leakiness of the xylose-inducible system is problematic for some essential genes, the vanillate-inducible system will likely be a good alternative.

The ease of generating CRISPRi-based knockdowns in Caulobacter crescentus should now enable a variety of whole-genome studies including systematic knockdowns of every gene, as has been done for B. subtilis (15). Large-scale transposon screens, e.g., transposon insertion sequencing (Tn-Seq), have been done with C. crescentus (29), but such studies may not cover all genes, depending on the size of the mutant library. Transposon-based approaches also cannot, in contrast to CRISPRi, investigate essential genes or target multiple genes. The ability to simultaneously target multiple genes will now enable systematic genetic interaction studies, a powerful approach for dissecting gene function that has previously been difficult to pursue at a global level in *Caulobacter*. As a proof of principle, we demonstrated that CRISPRi could be used to target both *cpaA* and *blaA*, producing cells lacking both pili and β-lactamase activity, which manifest as phage ΦCbK resistance and carbenicillin sensitivity.

One important limitation to the CRISPRi system is that it relies on the availability of unique targeting regions adjacent to a specific PAM site within, or near, the promoter region of the targeted gene. Although 20-nucleotide sequences are typically used as the targeting region in an sgRNA, it has been previously shown that the 12 nucleotides immediately upstream from the dCas9 handle are sufficient to recruit dCas9 (5, 13), increasing the probability of undesired binding elsewhere in the genome. We systematically looked for S. thermophilus PAM sequences in the *Caulobacter* genome that are unique and that target the nontemplate strand near the 5′ end of transcripts. We found that 96.5% of the genes with immediately preceding annotated transcriptional start sites (30) can be targeted with the S. thermophilus CRISPRi system developed here (see Table S2 in the supplemental material). The S. *pasteurianus* CRISPRi system increases the possibilities for sequences to target as it has a different PAM sequence and does not absolutely require its consensus PAM sequence (31). Together, the S. thermophilus and *S. pasteurianus* systems (using the NNGTGA PAM site) cover 97.1% of all genes immediately downstream of annotated transcriptional start sites (Table S2).

10.1128/mBio.02415-19.5 TABLE S2 Analysis of sgRNA target sequences. Download Table S2, DOCX file, 0.02 MB. Copyright © 2020 Guzzo et al. 2020 Guzzo et al. This content is distributed under the terms of the Creative Commons Attribution 4.0 International license.

We anticipate that the S. thermophilus CRISPR3 and *S. pasteurianus* dCas9 enzymes may also enable the development of CRISPRi systems in other alphaproteobacteria. As noted, the S. pyogenes dCas9 did not work in our hands for C. crescentus. This nonfunctionality may simply arise from insufficient expression owing, for instance, to suboptimal codon usage; alternatively, S. pyogenes dCas9 may be incompatible with some endogenous factor in C. crescentus and possibly other alphaproteobacteria. Whatever the case, our work reveals two functional dCas9 enzymes that can now be used to perform efficient and specific gene knockdowns in *Caulobacter* and possibly related organisms. This tool opens the door to a range of powerful genetic approaches that can be used to interrogate the biology of *Caulobacter*, including its cell cycle and hallmark cellular asymmetry.

## MATERIALS AND METHODS

### Growth conditions.

Caulobacter crescentus strains were grown in PYE (rich medium) at 30°C unless otherwise noted. Expression from the P*_xyl_* promoter was repressed with glucose (0.2%) and induced by supplementation with xylose (0.3%). Expression from the P*_van_* promoter was induced with vanillate (500 μM). When necessary, antibiotics were added at the concentrations shown in parentheses: kanamycin (5 μg/ml in liquid, 25 μg/ml in plates) and tetracycline (1 μg/ml in liquid, 2 μg/ml in plates).

### Strain construction.

All strains used are listed in Table S3 in the supplemental material. The CB15N strain of *Caulobacter* was used as the wild-type strain background for all strain constructions in this study. The insertion of *dcas9* (from the different organism system *Spy* [S. pyogenes], *Sth3*, or *Spa* [*S. pasteurianus*]) into the *Caulobacter* genome was done via homologous recombination at the *xylX* locus. The plasmid pXGFP-5-*dcas9* (or pVCERC-1-*dcas9*) was electroporated into wild-type competent cells, and recombinants were selected on PYE plates supplemented with tetracycline and glucose (or tetracycline only) to repress *dcas9* expression. The replicative high-copy-number plasmids bearing an sgRNA were transformed by electroporation, and transformants were selected on PYE plates supplemented with kanamycin.

10.1128/mBio.02415-19.6 TABLE S3 Strains, plasmids, and primers used. Download Table S3, DOCX file, 0.02 MB. Copyright © 2020 Guzzo et al. 2020 Guzzo et al. This content is distributed under the terms of the Creative Commons Attribution 4.0 International license.

### Design of CRISPR sgRNAs.

sgRNAs were designed using custom scripts in Python 2.7.6. For each PAM sequence (e.g., NGGNG), we first identified all instances of the sequence in the Caulobacter crescentus genome and then extracted the 20 bases ending 5′ of the PAM sequence as potential targeting sequences. To target a specific transcript for repression, we searched for targeting sequences in a 150-bp window from −50 to +100 of the transcriptional start site(s) directly preceding the first annotated coding region (30). Finally, we excluded any sgRNAs with potential off-target effects by excluding sgRNAs with seed regions (the 12 bp of the sgRNA adjacent to the PAM site) that had perfect complementarity next to multiple PAM sites elsewhere in the genome. Chosen sgRNAs were then cloned into the sgRNA expression vector as described below.

To calculate the number of genes and operons that can be targeted, we determined the number of genes and/or operons associated with at least one annotated transcriptional start site (30) and the number of unique nontemplate strand sgRNAs targeting those genes and/or operons. Unique sgRNAs were defined as sgRNAs whose 12-nt seed region was found only once in the genome next to an appropriate PAM site. Many genes have short 5′ untranslated regions and some nontemplate strand sgRNAs can be found after the translational start site; we have not tested the efficacy of sgRNA targeting of such regions. All *Spa* and *Sth3* nontemplate-strand sgRNAs targeting *Caulobacter* genes and/or operons are reported in Table S4.

10.1128/mBio.02415-19.7 TABLE S4 List of all *Caulobacter* nontemplate-strand sgRNAs. Download Table S4, XLSX file, 0.6 MB. Copyright © 2020 Guzzo et al. 2020 Guzzo et al. This content is distributed under the terms of the Creative Commons Attribution 4.0 International license.

### Plasmid construction.

All primers used are listed in Table S3, as are all plasmids used, which are also available for request via Addgene.

The plasmid carrying *dcas9* from S. pyogenes was constructed by first amplifying the *dcas9* gene (without codon optimization) from pdCas9 bacteria with primers dcas9pXGFP_up_F (*Spy*) and dcas9pXGFP_down_R (*Spy*) and amplifying the plasmid pXGFPC-5 (containing the P*_xyl_* promoter and *xylX* ribosome binding site) using primers dcas9pXGFP_up_R (*Spy*) and dcas9pXGFP_down_F (*Spy*). The *dcas9* insert and the amplified plasmid were cloned together using Gibson assembly.

The integration vectors carrying *dcas9* from *S. pasteurianus* and S. thermophilus CRISPR3 were cloned by PCR amplification of either the *Spa* or *Sth3 dcas9* from plasmid J468 or J663, respectively (16), with primers 781 and 782. The pXGFP-C plasmid was amplified with primers 783 and 784. The linear pieces were mixed and cloned using Gibson assembly.

The sgRNA was cloned into the pBXMCS-2 vector by first PCR amplifying the vector with primers 797 and 799 or 801 for *Spa* and *Sth3*, respectively. This amplification removed the *xylR* operator site and placed the transcriptional start site at the 5′ end of the 20-bp targeting sequence, thus leading to constitutive expression of the sgRNA. The dCas9 handle and transcriptional terminators were PCR amplified from plasmid JR468 or JR663 (16) with primers 796 and 798 or 800 for the *Spa* or *Sth3* sgRNA, respectively. The vector and sgRNA products were then cloned together using Gibson assembly.

For *ctrA* and *gcrA* sgRNA constructions, guide homology sequences were cloned into the modified pBXMCS-2 plasmid using round-the-horn PCR using primers XylA_R and the following forward primer depending on the construction: ctrA6_Sth3 (*sgRNA_ctrA* [*Sth3*]), gcrA_CRISPR_1 (*sgRNA_gcrA1* [*Sth3*]), gcrA_CRISPR_2 (*sgRNA_gcrA_2* [*Sth3*]), ctrA_Spas_F (*sgRNA_ctrA* [*Spa*]), and gcrA_Spas_F (*sgRNA_gcrA* [*Spa*]). The PCR products were then ligated and transformed into a cloning strain.

For the construction of the individual sgRNA (*Sth3*) plasmids to target the *blaA* or *cpaA* gene, complementary single-stranded ultramers were annealed using the following program from IDT: heat at 94°C for 3 min and then cool to 25°C over 45 min at a pace of 1.5°C per min. The modified pBXMCS-2 plasmid was linearized by PCR using primers CRISPRplasmid_F and CRISPRplasmid_R. The ultramers and linearized plasmid were cloned together using Gibson assembly. For the construction of the *cpaA* plus *blaA*, or *blaA* plus *cpaA*, plasmid used for the dual targeting, the plasmid carrying the sgRNA (*Sth3*) with the *cpaA*, or *blaA* targeting sequence, respectively, was amplified using primers dual_plasmid_dwn_F and dual_plasmid_up_R and the insert carrying the sgRNA (*Sth3*) with the *blaA*, or *cpaA* targeting sequence, respectively, was amplified using primers dual_insert_up_F and dual_insert_short_dwn_R. The two PCR products were cloned together using Gibson assembly.

### Serial dilution plating viability assay.

Strains were grown in PYE with appropriate antibiotics to an OD_600_ of ∼0.2 and then 10-fold serially diluted. Ten microliters of each dilution was spotted onto PYE plates containing, when appropriate, 0.2% glucose, 0.3% xylose, or 500 μM vanillate. Plates were incubated at 30°C for 2 days and imaged with a FluorChem R imager (ProteinSimple).

### Carbenicillin resistance assay.

Strains were grown overnight in PYE supplemented with kanamycin and 0.2% glucose. The following day, 200 μl of each culture was mixed with 3 ml of 0.5% top agar and poured onto a corresponding PYE plate. Both 0.5% and 1.5% agar PYE were supplemented with kanamycin and the indicated inducer (0.2% glucose or 0.3% xylose). Whatman paper 20-mm disks were soaked with 20 μl of a 10-mg/ml stock of carbenicillin, and three were placed on each plate. Plates were incubated overnight at 30°C. The diameters of growth inhibition were measured manually using Fiji and normalized to the average diameter (from the 3 disks per plate) of the wild type with empty vector on glucose or xylose.

### Phage sensitivity assays.

Phage sensitivity assays were performed to evaluate the resistance of different strains to the bacteriophage ΦCbK. First, to make a lawn of C. crescentus cells, 200 μl of stationary-phase cultures was mixed with 3 ml of 0.5% agar PYE and poured on a 1.5% PYE plate. Both 0.5% and 1.5% agar PYE were supplemented with kanamycin and the indicated inducer (0.2% glucose or 0.3% xylose). After the 0.5% agar PYE solidified, 5 μl of different dilutions of the phage ΦCbK (10^0^ to 10^−7^) in PYE was spotted on top. Plates were incubated for 2 days at 30°C before imaging.

### Time courses and synchronization.

When testing CRISPRi efficiency on mixed population of cells, strains were grown overnight in PYE supplemented with appropriate antibiotics and glucose (0.2%) and then diluted to an OD_600_ of ∼0.025 to 0.05. When cultures reached an OD_600_ of ∼0.1, they were split into two flasks, and at *t* = 0, one flask was supplemented with 0.3% xylose to induce the CRISPRi system. Note that the glucose was not washed away; xylose was added to the cultures already containing 0.2% glucose. At indicated time points, samples were harvested from each flask for flow cytometry (0.15 ml), immunoblotting (1 ml), RNA extraction followed by qRT-PCR (2 ml), and microscopy (1 ml). For flow cytometry, samples were stored in 30% ethanol at 4°C. For microscopy, samples were fixed with 0.5% paraformaldehyde, pelleted, resuspended in 1× PBS, and stored at 4°C. For immunoblotting and RNA extraction, cells were centrifuged for 1 min at 15,000 rpm, aspirated, and frozen in liquid nitrogen.

For the *ctrA401^ts^* strain, cells were grown to an OD_600_ of ∼0.1 and split into two flasks. One flask was maintained at 30°C, and the other was placed at the restrictive temperature of 37°C. Samples were harvested every 20 min for 2 h for flow cytometry (0.15 ml stored at 4°C in 30% ethanol), and after 2 h, samples were harvested for microscopy (1 ml).

For the GcrA depletion strain, cells were grown in PYE with 500 μM vanillate, kanamycin, and tetracycline to an OD_600_ of ∼0.1, centrifuged at 10,000 × *g* for 10 min, washed 3 times with PYE, released into PYE with kanamycin and tetracycline, and supplemented (or not) with 500 μM vanillate. Samples were harvested after 2 h for microscopy (1 ml).

When testing CRISPRi efficiency on synchronized populations of cells, strains were grown overnight in PYE supplemented with appropriate antibiotics and glucose (0.2%) and then diluted to an OD_600_ of ∼0.1 and grown to mid-exponential phase (OD_600_ of ∼0.25 to 0.4). Cells were then split into separate flasks. For cells induced presynchronization, they were washed free of glucose, resuspended in medium with xylose for the times indicated, and then synchronized. For all other cases, cells were grown in glucose until synchronization and then were released into the medium indicated. For synchronization, cells were first centrifuged for 10 min at 10,000 × *g*. G_1_/swarmer cells were isolated using Percoll (GE Healthcare) density gradient centrifugation. Briefly, pellets were resuspended in equal amounts of M2 buffer (0.87 g/liter Na_2_HPO_4_, 0.53 g/liter KH_2_PO_4_, 0.5 g/liter NH_4_Cl) and Percoll and centrifuged at 10,000 × *g* for 20 min. The upper ring was aspirated, and the lower ring, corresponding to swarmer cells, was transferred into a new 15-ml Falcon tube. Swarmer cells were washed in 13 ml of M2 buffer and centrifuged for 5 min at 10,000 × *g*. The pellet was resuspended in 2 ml M2 buffer and centrifuged for 1 min at 21,000 × *g*. Cells were released into PYE supplemented with appropriate antibiotics and glucose and/or xylose inducers.

For the RNA-seq and DNA microarray experiments, cells were grown in PYE supplemented with appropriate antibiotics and glucose (0.2%) overnight and then diluted to an OD_600_ of ∼0.025. When cultures reached an OD_600_ of ∼0.05 to 0.1, they were split into two flasks, and at *t* = 0, one flask was supplemented with 0.3% xylose to induce *dcas9* expression and the other remained in 0.2% glucose. Cells were grown for 2 h before 2-ml samples were harvested for RNA extraction.

### Reverse transcription coupled to quantitative PCR.

RNA was extracted using hot TRIzol lysis and the Direct-zol RNA miniprep kit (Zymo). A 2.5-μl amount of RNA at 100 ng/μl was mixed with 0.5 μl of 100-ng/μl random hexamer primers (Invitrogen), 0.5 μl of 10 mM deoxynucleoside triphosphates (dNTPs) and 3 μl of diethylpyrocarbonate (DEPC) water; incubated at 65°C for 5 min; and then placed on ice for 1 min. Two microliters of first-strand synthesis buffer, 0.5 μl of 100 mM dithiothreitol (DTT), 0.5 μl of SUPERase-In (ThermoFisher), and 0.5 μl of Superscript III (ThermoFisher) were added to each tube, and the following thermocycler program was used: 10 min at 25°C, 1 h at 50°C, and 15 min at 70°C. One microliter of RNase H (New England Biolabs [NEB]) was added, and each reaction mixture was incubated at 37°C for 20 min.

cDNA solutions were diluted 10 times in nuclease-free water for quantitative PCR (qPCR). One microliter of diluted cDNA or serially diluted genomic DNA (gDNA) used as a standard curve was mixed with an appropriate pair of primers, i.e., either *rpoA_qPCR_1* and *rpoA_qPCR_2* as a control, *ctrA_qPCR_1* and *ctrA_qPCR_5*, or *gcrA_qPCR_7* and *gcrA_qPCR_8.* All experimental samples were loaded as duplicates and with standard curves on a 384-well plate for qPCR. qPCR was conducted in a LightCycler 480 system (Roche) using the following thermocycler program: 95°C for 10 min, 95°C for 15 s, 60°C for 30 s, and 72°C for 30 s with 40 cycles of steps 2 to 4. Crossing point (*C_p_*) values were calculated from LightCycler 480 software at the second derivative maximum. Technical replicates were averaged to yield a final *C_p_* value for each sample and normalized to the standard curves. Each time point value for *ctrA* or *gcrA* was normalized to the *rpoA* measured value, as *rpoA* expression remains constant in exponential phase.

### Immunoblotting.

Frozen pellets from the time course sampling were normalized by OD for resuspension in 1× blue loading buffer (NEB) supplemented with 1× reducing agent (DTT), boiled at 95°C for 10 min, and loaded on 12% gels (Bio-Rad) for electrophoresis. Proteins were transferred from the gel into polyvinylidene difluoride (PVDF) membranes and immunoblotted. Antibodies were used at the concentrations shown in parentheses: anti-RpoA (1:5,000, BioLegend), anti-CtrA (1:5,000), and anti-GcrA (1:5,000). Horseradish peroxidase (HRP)-conjugated secondary antibodies (ThermoFisher) were used at the concentrations shown in parentheses: anti-mouse (1:10,000) and anti-rabbit (1:5,000). The membranes were developed with SuperSignal West Femto maximum-sensitivity substrate (ThermoFisher) and visualized with a FluorChem R Imager (ProteinSimple). RpoA immunoblotting was used at the loading control for each sample. Protein band intensities were measured using Fiji.

### Flow cytometry.

A fraction of fixed cells from the time course sampling (corresponding to an OD_600_ of ∼0.005) were centrifuged at 6,000 rpm for 4 min. Pelleted cells were resuspended in 1 ml of Na_2_CO_3_ buffer containing 3 μg/ml RNase A (Qiagen) and incubated at 50°C for at least 4 h. Cells were supplemented with 0.5 μl/ml SYTOX Green nucleic acid stain (Invitrogen) in Na_2_CO_3_ buffer and analyzed on a MACSQuant VYB flow cytometer. Data were analyzed with FlowJo software.

### Microscopy.

Fixed cells from the time courses were concentrated to an OD_600_ of ∼0.4. One microliter of cells was spotted onto PBS-1.5% agarose pads and imaged. Phase-contrast images were taken on a Zeiss Observer Z1 microscope using a 100×/1.4 oil immersion objective and an LED-based Colibri illumination system using MetaMorph software (Universal Imaging, PA).

### Analysis of RNA-seq and microarray data.

RNA-seq libraries were sequenced by paired-end sequencing on an Illumina NextSeq sequencer at the MIT BioMicro Center. Data analysis was performed using custom scripts written in Python 2.7.6. Sequencing reads were aligned to *Caulobacter* NC011916.1 with Bowtie 2 (version 2.1.0) using the default parameters. SAMtools (version 0.1.19) was used with the pysam library (version 0.9.1.4) for conversion between BAM and SAM file formats and indexing reads. The read coverage was mapped to the *Caulobacter* genome by assigning each mapped base a value of 1/*N* where *N* equals the length from the 5′ end to the 3′ end of each paired read. To calculate mRNA abundance, a pseudocount was added to all positions and the number of reads mapped to a gene was divided by the length of the gene and normalized to yield the mean number of reads per kilobase per million sequencing reads (RPKM). The change in gene expression was calculated by taking the log_2_-RPKM ratio of each gene from the experimental condition to the control condition (*dcas9* grown in xylose/*dcas9* grown in glucose; sgRNA-*ctrA* grown in xylose/sgRNA-*ctrA* grown in glucose). To identify genes differentially expressed in xylose compared to glucose, we calculated the log_2_-RPKM ratio for each gene from wild-type cells grown in xylose to wild-type cells grown in glucose (32). DNA microarray experiments were performed and analyzed as reported previously (23).

### Data availability.

Expression data were deposited in GEO (GSE139521).
