# Supplementary figures and images for: A CRISPR Interference System for Efficient and Rapid Gene Knockdown in Caulobacter crescentus
Source: mBio. 2020 Jan 14;11(1):e02415-19. doi: 10.1128/mBio.02415-19 (PMC6960281; doi:10.1128/mBio.02415-19)

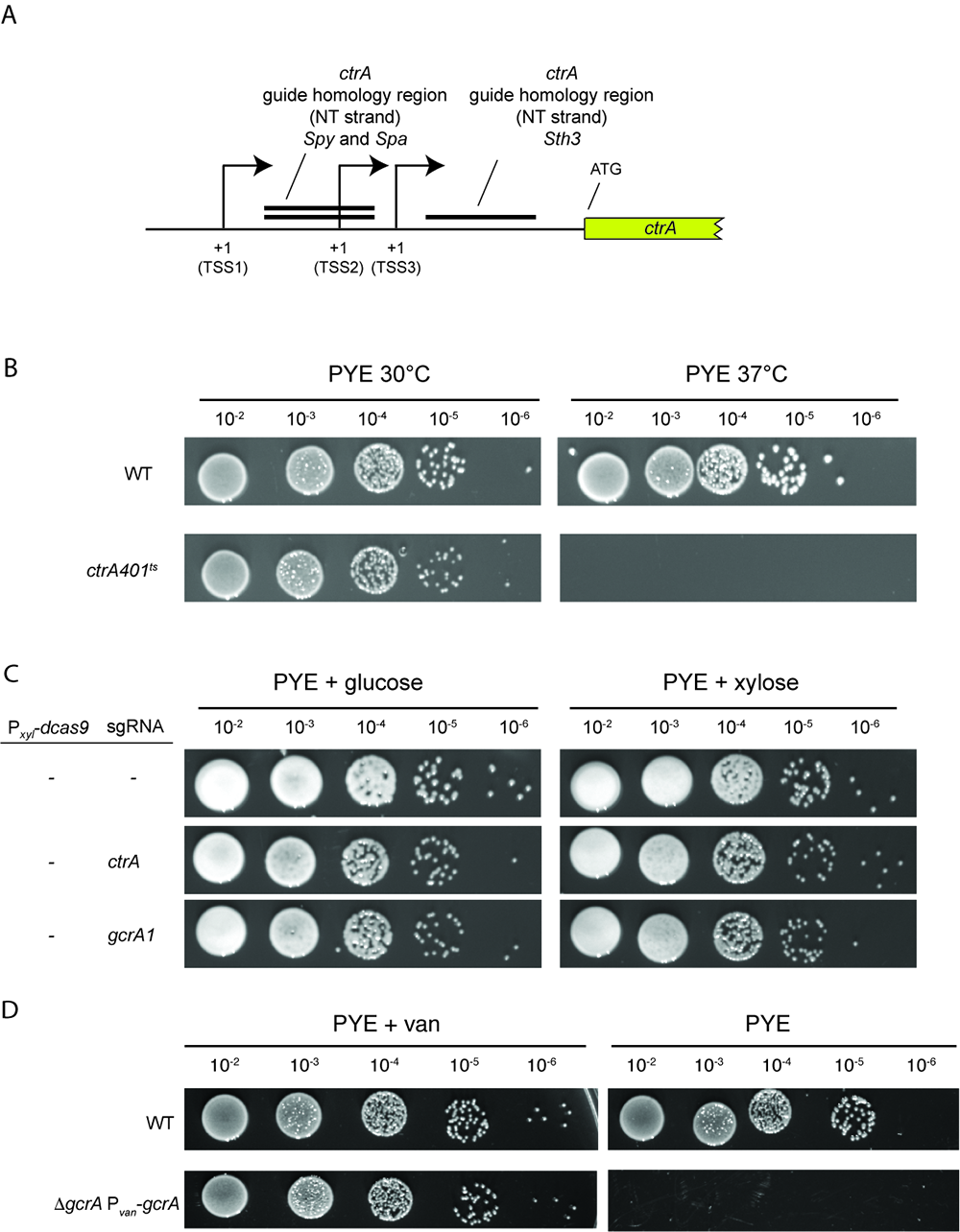

Supplement: FIG S1 [file mBio.02415-19-sf001.tif]

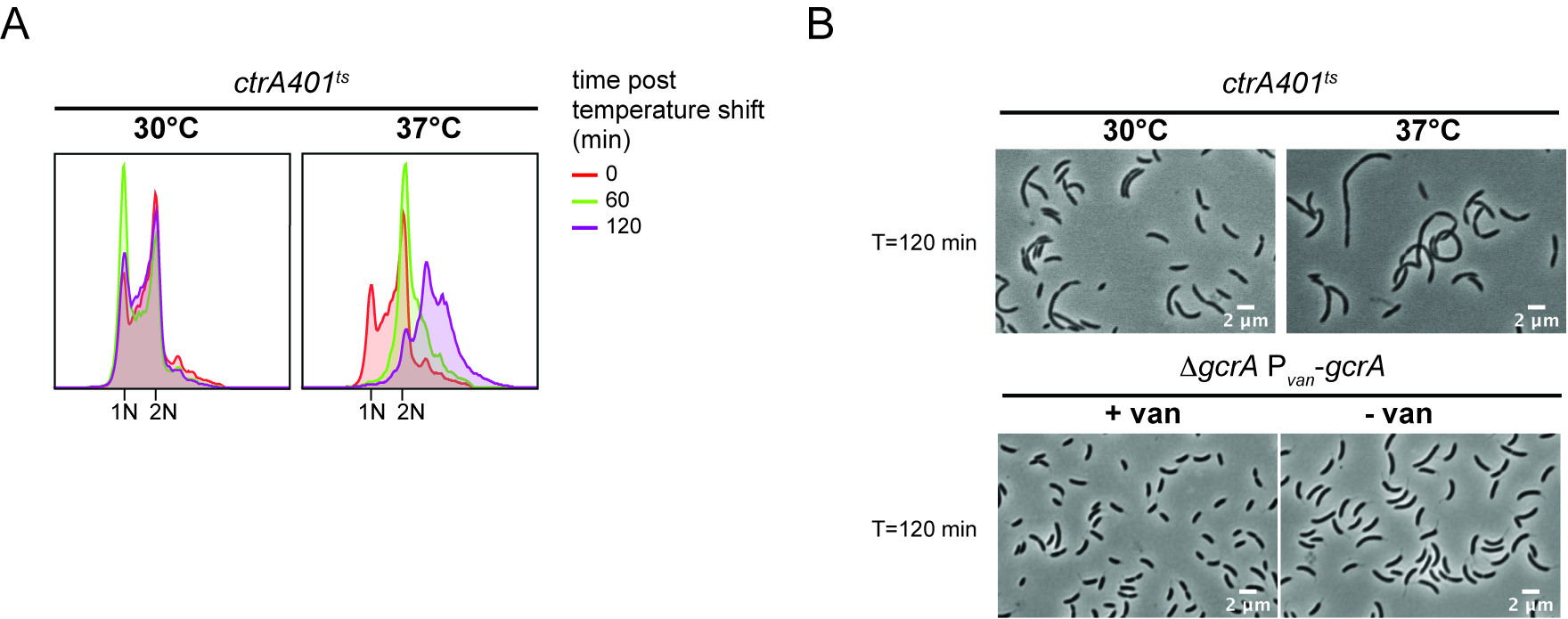

Supplement: FIG S2 [file mBio.02415-19-sf002.tif]

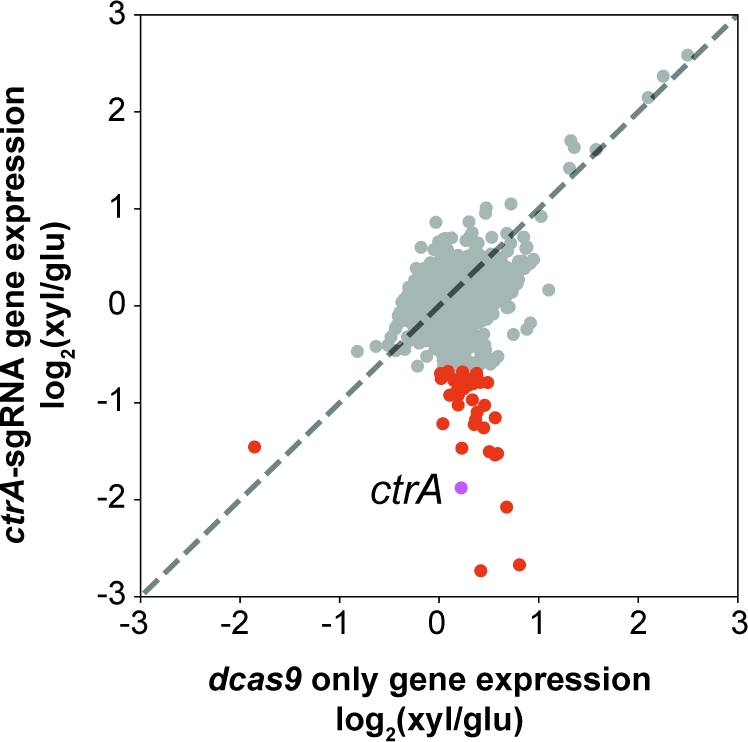

Supplement: FIG S3 [file mBio.02415-19-sf003.tif]
