# Supplementary material for: A CRISPR Interference System for Efficient and Rapid Gene Knockdown in Caulobacter crescentus
Source: mBio. 2020 Jan 14;11(1):e02415-19. doi: 10.1128/mBio.02415-19 (PMC6960281; doi:10.1128/mBio.02415-19)
Supplement: TABLE S2 [file mBio.02415-19-st002.docx]

**Table S2 – Analysis of sgRNA target sequences.**

| **# of Transcription Start Sites^1^ (TSS)** | 2,726 |
| --- | --- |
| **# of gene-associated TSS^2^** | 2,662 |
| **# of TSS-associated gene/operon units (GU)^3^** | 1,878 |

| ***Streptococcus thermophilus* CRISPR3** | | **264,782 guides** | |
| --- | --- | --- | --- |
| GU w/o PAM site | 0 | GU w/ PAM site | 1,878 |
| GU w/o NTS^4^ sgRNA | 48 | GU w/ NTS sgRNA | 1,830 |
|  |  | **GU w/ unique NTS sgRNA^5^** | **1,813 (96.5%)** |
|  | | | |
| ***Streptococcus pasteurianus*** | | **25,334 guides** | |
| GU w/o PAM site | 668 | GU w/ PAM site | 1,210 |
| GU w/o NTS sgRNA | 1,495 | GU w/ NTS sgRNA | 383 |
|  |  | **GU w/unique NTS sgRNA^5^** | **379 (20.2%)** |
|  | | | |
| ***S. pasteurianus + S. thermophilus*** | | **290,116 guides** | |
| GU w/o PAM site | 0 | GU w/ PAM site | 1,878 |
| GU w/o NTS sgRNA | 42 | GU w/ NTS sgRNA | 1,836 |
|  |  | **GU w/unique NTS sgRNA^5^** | **1,823 (97.1%)** |

**^1^**Transcription Start Sites from Zhou and Schrader et al, *PLoS Genet*., 2015.

**^2^**Number of TSS associated with annotated genes.

**^3^**Number of unique TSS-associated genes and/or operon units (GU). GU are defined as number genes/operons found with any annotated TSS site (i.e. *ctrA* has 3 TSS but is counted once).

^4^NTS = non-template strand.

**^5^**Number of unique sgRNAs targeting genes and/or operons. Unique sgRNA is defined as an sgRNA with a 12 nt seed region that has only one perfect complement in the chromosome with an appropriate PAM site. The total number of GU and the percentage of GU that can be targeted are reported.
