# Supplementary material for: A CRISPR Interference System for Efficient and Rapid Gene Knockdown in Caulobacter crescentus
Source: mBio. 2020 Jan 14;11(1):e02415-19. doi: 10.1128/mBio.02415-19 (PMC6960281; doi:10.1128/mBio.02415-19)
Supplement: TABLE S3 [file mBio.02415-19-st003.docx]

**Table S3 - Strains, Plasmids, and Primers**

| ***Caulobacter crescentus* strains** | |  |
| --- | --- | --- |
| ML76 | CB15N WT | Lab collection |
| ML79 | CB15N *ctrA401^ts^* | Quon et al 1998 |
| ML2302 | CB15N Δ*gcrA* P*_van_*-*gcrA* | Haakonsen et al 2015 |
| ML3171 | CB15N P*_xyl_*-*dcas9* (*Spy*) | This study |
| ML3172 | CB15N P*_xyl_*-*dcas9* (*Spy*) P*_constitutive_-sgRNA(Spy)_ctrA* | This study |
| ML3173 | CB15N P*_xyl_*-*dcas9* (*Sth3*) | This study |
| ML3174 | CB15N P*_xyl_*-*dcas9* (*Sth3*) P*_constitutive_*-*sgRNA(Sth3)_ctrA* | This study |
| ML3175 | CB15N P*_xyl_*-*dcas9* (*Sth3*) P*_constitutive_*-*sgRNA(Sth3)_gcrA1* | This study |
| ML3176 | CB15N P*_xyl_*-*dcas9* (*Sth3*) P*_constitutive_*-*sgRNA(Sth3)_gcrA2* | This study |
| ML3177 | CB15N P*_xyl_*-*dcas9* (*Spa*) | This study |
| ML3178 | CB15N P*_xyl_*-*dcas9* (*Spa*) P*_constitutive_*-*sgRNA(Spa)_ctrA* | This study |
| ML3179 | CB15N P*_xyl_*-*dcas9* (*Spa*) P*_constitutive_*-*sgRNA(Spa)_gcrA3* | This study |
| ML3180 | CB15N P*_van_*-*dcas9* (*Sth3*) | This study |
| ML3181 | CB15N P*_van_*-*dcas9* (*Sth3*) P*_constitutive_*-*sgRNA(Sth3)_ctrA* | This study |
| ML3182 | CB15N P*_constitutive_*-*sgRNA(Sth3)_ctrA* | This study |
| ML3183 | CB15N P*_constitutive_*-*sgRNA(Sth3)_gcrA1* | This study |
| ML3184 | CB15N P*_xyl_*-*dcas9* (*Sth3*) P*_constitutive_*-*sgRNA(Sth3)_cpaA* | This study |
| ML3185 | CB15N P*_xyl_*-*dcas9* (*Sth3*) P*_constitutive_*-*sgRNA(Sth3)_blaA* | This study |
| ML3186 | CB15N P*_xyl_*-*dcas9* (*Sth3*) P*_constitutive_*-*sgRNA(Sth3)_cpaA-* P*_constitutive_*-*sgRNA(Sth3)_blaA* | This study |
| ML3187 | CB15N P*_xyl_*-*dcas9* (*Sth3*) P*_constitutive_*-*sgRNA(Sth3)_blaA-* P*_constitutive_*-*sgRNA(Sth3)_cpaA* | This study |

| **Plasmids** |  | Addgene ID |  |
| --- | --- | --- | --- |
| ML3188 | DH5α pXGFPC-5 P*_xyl_*-*dcas9* (*Spy*) | 133316 | This study |
| ML3189 | Top10 pXGFPC-5 P*_xyl_*-*dcas9* (*Sth3*) | 133317 | This study |
| ML3190 | Top10 pXGFPC-5 P*_xyl_*-*dcas9* (*Spa*) | 133318 | This study |
| ML3191 | Top10 pVCERC-1 P_van_-*dcas9* (*Sth3*) | 133319 | This study |
| ML3192 | DH5α pBXMCS-2 P*_constitutive_*-*sgRNA(Spy)_ctrA* | 133334 | This study |
| ML3193 | Top10 pBXMCS-2 P*_constitutive_*-*sgRNA(Sth3)_ctrA* | 133339 | This study |
| ML3194 | Top10 pBXMCS-2 P*_constitutive_*-*sgRNA(Sth3)_gcrA1* | 133340 | This study |
| ML3195 | Top10 pBXMCS-2 P*_constitutive_*-*sgRNA(Sth3)_gcrA2* | 133341 | This study |
| ML3196 | DH5α pBXMCS-2 P*_constitutive_*-*sgRNA(Spa)_ctrA* | 133342 | This study |
| ML3197 | DH5α pBXMCS-2 P*_constitutive_*-*sgRNA(Spa)_gcrA3* | 133343 | This study |
| ML3198 | DH5α pBXMCS-2 P*_constitutive_*-*sgRNA(Sth3)_cpaA* | 133344 | This study |
| ML3199 | DH5α pBXMCS-2 P*_constitutive_*-*sgRNA(Sth3)_blaA* | 133345 | This study |
| ML3200 | DH5α pBXMCS-2 P*_constitutive_*-*sgRNA(Sth3)_cpaA*- P*_constitutive_*-sgRNA*(Sth3)*_*blaA* | 133347 | This study |
| ML3201 | DH5α pBXMCS-2 P*_constitutive_*-*sgRNA(Sth3)_blaA*- P*_constitutive_*-sgRNA(*Sth3*)_*cpaA* | 133348 | This study |

| **Primers** |  |  |
| --- | --- | --- |
|  | AGCTCCTGACTCAGGAGGTATCGATATGGATAAGAAATACTCAATAGGCT | dcas9pXGFP_up_F (Spy) |
|  | AGCCTATTGAGTATTTCTTATCCATATCGATACCTCCTGAGTCAGGAGCT | dcas9pXGFP_up_R (Spy) |
|  | TTTGAGTCAGCTAGGAGGTGACTAAATACTCTGGCCATTTTTCTTTACCG | dcas9pXGFP_down_F (Spy) |
|  | CGGTAAAGAAAAATGGCCAGAGTATTTAGTCACCTCCTAGCTGACTCAAA | dcas9pXGFP_down_R (Spy) |
|  | tctcgagctcCTGACTCaggaggtatcgat | 781 newCas9F |
|  | tggatcccccgggctgcagctagcAAGCGAAAAAGGCATCCG | 782 newCas9R |
|  | atcgatacctcctGAGTCAGgagctcgagatcttaaggtacc | 783 pXGFP R forcas |
|  | tgcagcccgggggatc | 784 pXGFP F forcas |
|  | TGAAAAAGTGGCACCGAGTCGGTGCTTTTGATAAAACGAAAGGCCCAGTC | sgRNASpyo_F |
|  | GCACCGACTCGGTGCCACTTTTTCAAGTTGATAACGGACTAGCCTTATTTTAACTTGCTATTTCTAGCTCTAAAACAAGTCTTCAGCTGC | sgRNASpyo_R |
|  | GCTAGCCCATGGgaattcctgcagcccgg | 797 pBXMCS F JR |
|  | GCCTGTTTCCGATCCTCCATgtcggcacttggtagcgc | 799Spas CcFtsA R |
|  | TCAGGCCTTCACGGGCCTGTgtcggcacttggtagcg | 801Sthe3 CcFtsA R |
|  | gaattcCCATGGGCTAGCGGCTT | 796 JR sgRNA R |
|  | ATGGAGGATCGGAAACAGGC GTTTTTGTACTCGAAAGAGCCT | 798Spas CcFtsA |
|  | ACAGGCCCGTGAAGGCCTGA GTTTTAGAGCTGTGAAAACAGCG | 800Sthe3 CcFtsA |
|  | GTCGGCACTTGGTAGCG | XylA_R |
|  | ACGTCTTTGGCGGGAGTCCGCgttttagagctgtgaaaacagc | ctrA6_STh3 |
|  | CACGCCCGTCACCCTATATAgttttagagctgtgaaaacagc | gcrA_CRISPR_1 |
|  | CGGATCGATCACCATATATAgttttagagctgtgaaaacagc | gcrA_CRISPR_2 |
|  | cgctaccaagtgccgacGTTAATTTAAGACTGGTTAAgtttttgtactcgaaagagccta | ctrA_Spas_F |
|  | cgctaccaagtgccgacCGGATCGATCACCATATATAgtttttgtactcgaaagagccta | gcrA_Spas_F |
|  | ACATCGTCTACATCGGCGAC | rpoA_qPCR_1 |
|  | GGCGAGCACTTCCTTGATCT | rpoA_qPCR_2 |
|  | GTACGACCCTGACCAAGGAA | ctrA_qPCR_1 |
|  | GCAGATGAAGACGTCGATGA | ctrA_qPCR_5 |
|  | ATGAGCTGGACCGACGAAC | gcrA_qPCR_7 |
|  | CCAATTGCTTGGCGATCTGG | gcrA_qPCR_8 |
|  | gttttagagctgtgaaaacagcgagttaaa | CRISPRplasmid_F |
|  | tcggcacttggtagcgctaacatgtgggga | CRISPRplasmid_R |
|  | tccccacatgttagcgctaccaagtgccgaGAGCGTGACGCGAGTGGCTTgttttagagctgtgaaaacagcgagttaaa | cpaAultramer_F |
|  | tttaactcgctgttttcacagctctaaaacAAGCCACTCGCGTCACGCTCtcggcacttggtagcgctaacatgtgggga | cpaAultramer_R |
|  | tccccacatgttagcgctaccaagtgccgaCCAGGATCAGGCGCTTCATGgttttagagctgtgaaaacagcgagttaaa | blaA1_ultramer_F |
|  | tttaactcgctgttttcacagctctaaaacCATGAAGCGCCTGATCCTGGtcggcacttggtagcgctaacatgtgggga | blaA1_ultramer_R |
|  | ttcctgcagcccgggggatccactagttctcaaataaaacgaaaggctcagtcgaaagac | dual_insert_up_F |
|  | agaactagtggatcccccgggctgcaggaa | dual_plasmid_up_R |
|  | ccaattcgccctatagtgagtcgtattacg | dual_plasmid_dwn_F |
|  | cgtaatacgactcactatagggcgaattggaagacccgtttataaaacgaaaggctcagt | dual_insert_short_dwn_R |
